# Revolutionizing cross-border e-commerce: A deep dive into AI and big data-driven innovations for the straw hat industry

**DOI:** 10.1371/journal.pone.0305639

**Published:** 2024-12-20

**Authors:** Junjie Dai, Xiaoyan Mao, Pengyue Wu, Huijie Zhou, Lei Cao

**Affiliations:** 1 College of Science and Technology, Ningbo University, Ningbo, Zhejiang, China; 2 Faculty of Electrical Engineering and Computer Science, Ningbo University, Ningbo, Zhejiang, China; Federal University of Goias: Universidade Federal de Goias, BRAZIL

## Abstract

This paper investigates the impact of artificial intelligence (AI) and big data analytics on optimizing cross-border e-commerce efficiency for straw hat manufacturers in Zhejiang Province, China. It identifies market and consumer demand trends through machine learning analysis of comprehensive e-commerce data and leverages generative AI to revolutionize production and marketing processes. The integration of AI-generated content (AIGC) technology facilitates streamlined design-to-production cycles and rapid adaptation to market changes and consumer feedback. Findings demonstrate that the application of AI and big data significantly enhances market responsiveness and sales performance for straw hat enterprises in cross-border e-commerce. This research contributes a novel framework for employing AI and big data to navigate the complexities of international commerce, providing strategic insights for small and micro enterprises seeking to expand their global market footprint.

## 1. Introduction

Straw hats from China, as an intangible cultural heritage that has been passed down for thousands of years, possess unique charms in both functionality and artistic appreciation. Made from materials like aquatic grass, wheat straw, or palm ropes, these straw hats, due to their environmentally friendly attributes, have gradually become the preferred choice among similar products for consumers, enjoying popularity among customers both domestically and internationally. Zhejiang Province, located along China’s southeast coast, with its abundant labor resources, advanced technology support, and convenient maritime shipping conditions, serves as a stronghold for the cross-border trade of straw hats. In Cixi City, Zhejiang Province alone, there are more than 50 straw hat manufacturing enterprises, with an annual output value of up to 250 million yuan; among these, the golden silk straw hats produced in Changhe Town are sold in nearly 70 countries and regions around the world.

However, due to reasons such as domestic overproduction and saturated demand, an increasing number of companies are shifting their sales focus overseas and selling their products through channels such as e-commerce. This shift, however, has introduced many new challenges. On one hand, rising labor costs continue to erode corporate profits and greatly hinder the efficiency of processing foreign trade orders. A simple example is that the increasing number of orders will continuously add to the number of personnel in the corresponding business modules, often involving demand docking, design, contract signing, etc.; this represents an unavoidable yet crucial expense for handicraft enterprises like those producing straw hats. On the other hand, e-commerce emphasizes the unique selling points of products, catering to mainstream demand, and creating best-selling items; however, small and micro enterprises often lack the technical conditions necessary, resulting in a lack of comprehensive understanding of the market and consumers. This makes the production of goods challenging, and may even lead to a situation where products accumulate in large quantities due to a sudden drop in popularity.

Indeed, enhancing the efficiency of cross-border e-commerce services and improving sales levels have always been hot topics [1–3]. With the acceleration of globalization and continuous advancements in internet technology, both enterprises and researchers are seeking effective methods to address the challenges encountered in the process of cross-border e-commerce, such as complex market research, significant cultural differences, and legal and regulatory restrictions. To tackle these challenges, a series of innovative strategies and technologies have been proposed and implemented. For instance, the use of big data and machine learning technologies to analyze consumer behavior and predict market trends allows for more precise production management and marketing strategies [4–6]. Natural Language Processing (NLP) technology analyzes the vast amount of textual data left by consumers online, including product reviews, social media posts, forum discussions, etc. This textual data often contains rich information about consumer sentiments, preferences, and needs. For e-commerce enterprises, effectively analyzing and utilizing this data can lead to accurate consumer group positioning and demand understanding [7, 8].

The focus of the above content is on both the market and consumers, revealing the trends and movements of both through data and algorithms. However, for enterprises, the ability to rapidly respond to orders is also a highly competitive feature in foreign trade [9]; especially in cross-border e-commerce, where consumer demand for products is becoming increasingly diverse and personalized. This requires enterprises to have the ability to quickly adapt to market changes, as well as the capacity to complete product design, production, and delivery in the shortest time possible, in order to cope with the global market environment.

The structure of this paper is as follows: Section 2 is a literature review related to the application of big data, machine learning, natural language processing, and artificial intelligence in the field of e-commerce. In Section 3, we will take the cross-border e-commerce of straw hat products as an example to elaborate on the impact of different product characteristics on the sales of straw hats and to develop a comprehensive sales strategy. Section 4 explores the application of generative artificial intelligence in handling cross-border orders for small and micro enterprises. Section 5 proposes a production and sales framework for cross-border e-commerce products suitable for most enterprises.

## 2. Literature review

Cross-border e-commerce, fundamentally a new branch of e-commerce, has gradually become a new external product sales channel under the influence of the increasingly saturated domestic e-commerce market [10–12]. Currently, over a billion people worldwide engage in cross-border online shopping, with the value of international e-commerce far exceeding 179 billion USD [13]; among which, China holds more than a third of the market share, making it the most attractive investment market at present.

E-commerce inherently facilitates data collection, and this data possesses concentrated business value. In the era of big data, the scope of information collection is continuously expanding, providing substantial assistance to e-commerce marketing and management activities. It shows exceptional performance in improving operational efficiency, promoting the development of personalized marketing activities, and broadening sales channels [14–17]. Big data encompasses detailed information on goods, users’ browsing history, or purchase records, among other things. Analyzing these data can provide merchants with a convenient channel to grasp market trends and focus on competition for the same goods. It also supports the precise positioning of customer profiles and the implementation of personalized marketing based on customer needs.

However, the challenge we face is not in obtaining data [18, 19], but in using the correct data to gain market knowledge through computing. Studies have indicated that most enterprises lack the means to collect and analyze data information, diminishing the application value of data in production and sales [20]. More scholars are applying various automation technologies in the field of e-commerce, including machine learning, natural language processing, and even the currently popular artificial intelligence. Machine learning technology is an efficient tool for learning from existing data, fitting models to perform tasks such as prediction and classification [21, 22]. By analyzing historical purchase data, user behavior patterns, and market trends, machine learning algorithms can accurately predict which styles of goods will be popular, thereby helping merchants to formulate more effective marketing strategies and inventory management plans. This not only improves customer satisfaction but also optimizes resource allocation and reduces the risk of inventory backlog [23]. Natural Language Processing (NLP) can understand semantics from a wide range of textual data, thus conducting sentiment analysis, entity recognition, and topic modeling, which helps in interpreting customer reviews and needs [24]. Li discussed the needs of adaptive clothing consumers through online reviews, using the LDA model to emphasize the importance of functional considerations, the desire for fashion products, and the value of a positive online shopping experience [25]; Zhuo proposed an enhanced LDA model using fuzzy set word vector technology to more accurately and effectively extract consumer demand information from product reviews [26]; Jabbar conducted real-time sentiment analysis in e-commerce applications, achieving a rapid response to consumer feedback and needs [27].

In recent years, the application of artificial intelligence-related technologies across various fields has achieved tremendous success [28–32], bringing unprecedented opportunities to e-commerce and cross-border trade. Zhu has facilitated real-time, barrier-free cross-border trade communication through AI-powered language translation [33]; Rui and colleagues have explored the use of image recognition technology in AI to classify cross-border e-commerce products, thereby enhancing the automation level of product classification and providing consumers with more accurate search results [34]. Aliyev and others have studied AI-based chatbots for the modernization of e-commerce and logistics platforms, aimed at improving operational efficiency and customer service quality [35]; Achar discussed the early impact of virtual assistants in international trade, including how to improve trade efficiency through automation [36]. Undoubtedly, as technology continues to advance and become more accessible, discussions around artificial intelligence will become more frequent, especially with the advent of generative AI (GAI) technologies like Chat-GPT and DALL-E in 2022, opening new perspectives and indicating a potential revolution in the operation of most industries. However, existing GAIs still face several key issues that need to be addressed, such as high training and maintenance costs, high data sparsity, poor integration of domain knowledge, issues with model interpretability and credibility, resource sharing, security, etc. [37–40]. These represent the preliminary challenges that need to be overcome for the application of GAI in cross-border e-commerce.

## 3. Method

### 3.1 Data sources and preprocessing

To understand the current market situation of straw hat sales on cross-border e-commerce platforms, we selected "Alibaba 1688" wholesale procurement website as our primary data source. Straw hats, as a common accessory in the fashion industry, predominantly utilize distribution as their main sales channel. Alibaba 1688 is a renowned brand in global business-to-business (B2B) e-commerce, offering millions of online merchants a wealth of business opportunities and a convenient and secure online transaction environment. This has resulted in the accumulation of vast amounts of valuable information, making the site an operable and effective source for our data analysis.

We searched for sales information on products using "straw hat" and "woven hat" as the main keywords. The search results page presented detailed information on each product, covering a rich array of product attributes such as "product name,""price," and "reviews." A Python 3.10 script was written to scrape the aforementioned data, yielding a total of 3,607 unique entries for the entire year of 2023. The data encompasses 187 key product features, with a partial display of the data shown in the Table 1:

**Table 1:** Partial data information display.

Serial number	Trade name	Service rating	Price	Collection volume	Sales volume	. . .
1	Sun Protection Hat Summer Large Brim Double-sided New Model Hollow Top Sunshade Cap Women UV Protection Ponytail Exposing Sun Hat	0.75	28	113	2582	. . .
2	Export-Specific Explorer Safety Anti-Mosquito Beekeeping Hat Vietnamese Helmet Straw Hat Sunshade Bucket Hat Factory Wholesale	0.83	28	490	27858	. . .
3	2023 Organza Bucket Hat Korean Version Butterfly Knot Mesh Sunshade Cap Women Summer Travel Sun Protection Sun Hat Cooling Hat	0.53	25	1713	13022	. . .
. . .	. . .	. . .	. . .	. . .	. . .	. . .

Given the large number of product features and the inconsistency in information provided by merchants, the data contained missing values and redundancies that required further decomposition and integration. For instance, "material" and "fabric" were considered to be the same attribute, while "processing technology" and "manufacturing method" were deemed to be the same production process. Moreover, some attribute columns had a significant amount of missing information, with valid entries constituting less than 5% (180 entries) of the total dataset. For the sake of data integrity, it was necessary to exclude these features. Additionally, when the same product was listed on e-commerce platforms by different merchants, the information fields filled out were not consistent. For example, the "applicable season" attribute with entries like "all seasons" and "spring, summer, autumn, winter" were considered to represent the same information. Therefore, it was necessary to standardize the data. After such processing, the number of product attributes was reduced to 49, retaining the majority of beneficial content.

### 3.2 Data analysis

#### 3.2.1 Overview of the straw hat industry

Based on the data collected, we conducted an analysis of the domestic straw hat industry, detailed as follows:

*(1) Main production and market areas*. The straw hat industry in China is primarily concentrated in the eastern region, with the top ten producing provinces, except for Henan and Shaanxi, mostly located in the East and South of China. Zhejiang province far exceeds other provinces in output, thus becoming a focal area of the straw hat industry. The city of Yiwu, known as the "Capital of Small Commodities" in China, alone accounts for more than a quarter of Zhejiang’s straw hat production orders. Zhejiang not only possesses the world’s largest port in terms of throughput but its developed transportation and convenient logistics system also provide strong support for the development of the straw hat industry. With such industrial and infrastructure support, Zhejiang’s import and export trade of straw hats ranks among the top nationwide.

With the flourishing of international trade, e-commerce platforms have become a crucial channel for straw hats to be sold around the globe. Against this backdrop, the international consumer market has become the focal point of attention for straw hat manufacturers. Table 2 indicates that the European and American regions are the primary markets for overseas consumption of straw hats, with sales figures leading the pack. The selling price across different regions does not vary much, maintaining a level of approximately 10 RMB per piece. It is important to note that the data reflects wholesale prices for straw hats destined for export, which differ from the actual retail prices. Further analysis regarding the actual selling prices will be conducted in subsequent sections.

**Table 2:** Sales of straw hats in major international markets.

Area	Total sales	Sales volume	Average selling price
North America	12517215	126275578.80	10.09
Europe	12701226	124992992.00	9.84
South America	12301160	121217575.90	9.85
Middle East	11331694	118194281.90	10.43
Southeast Asia	11961105	117457327.90	9.82
Northeast Asia	10841495	106911689.70	9.86
Africa	9495218	94002788.68	9.90
Japan and South Korea	1579295	16684010.91	10.56

**Fig 1:**
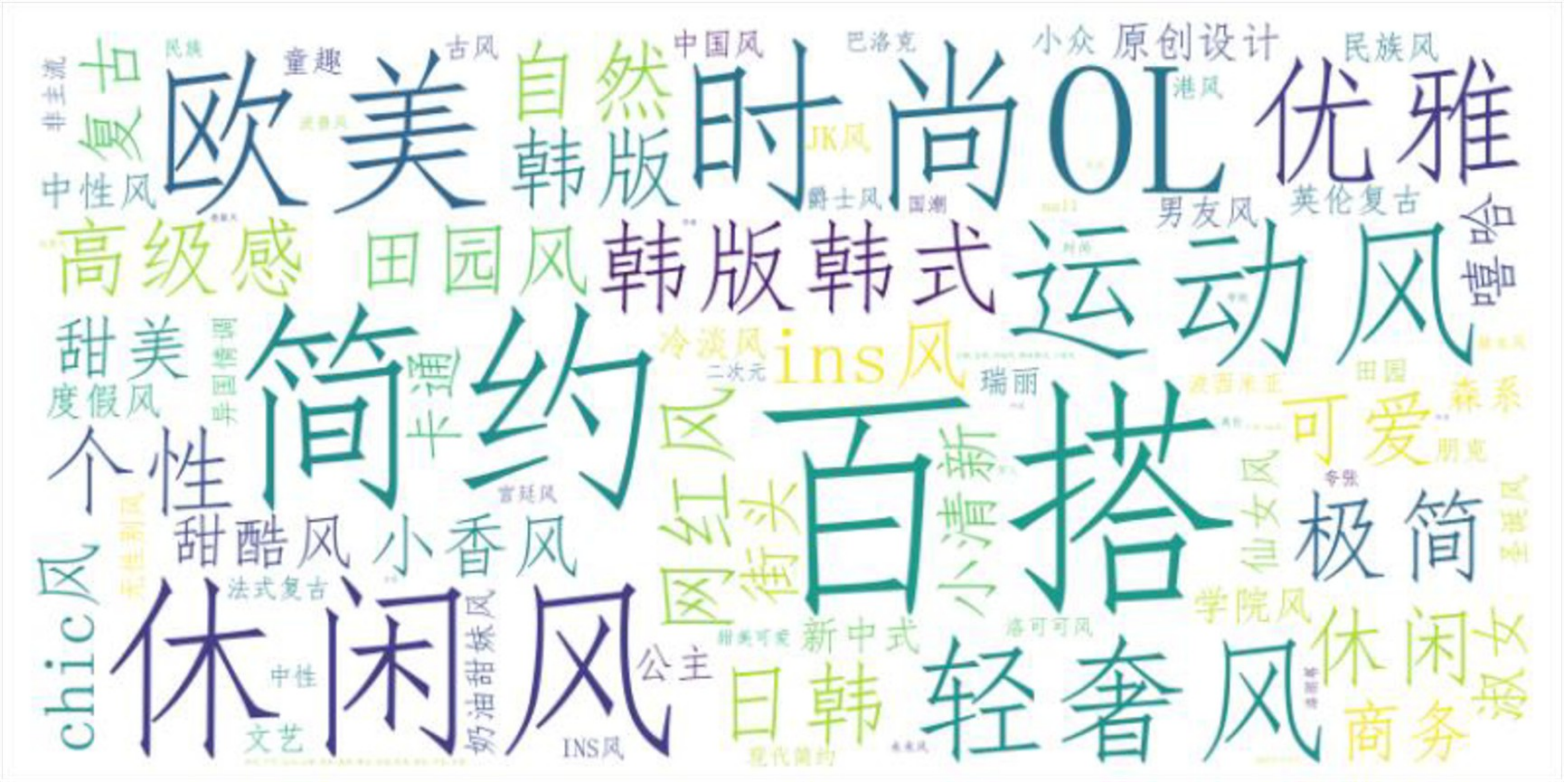
Straw hat product style word cloud.

**Fig 2:**
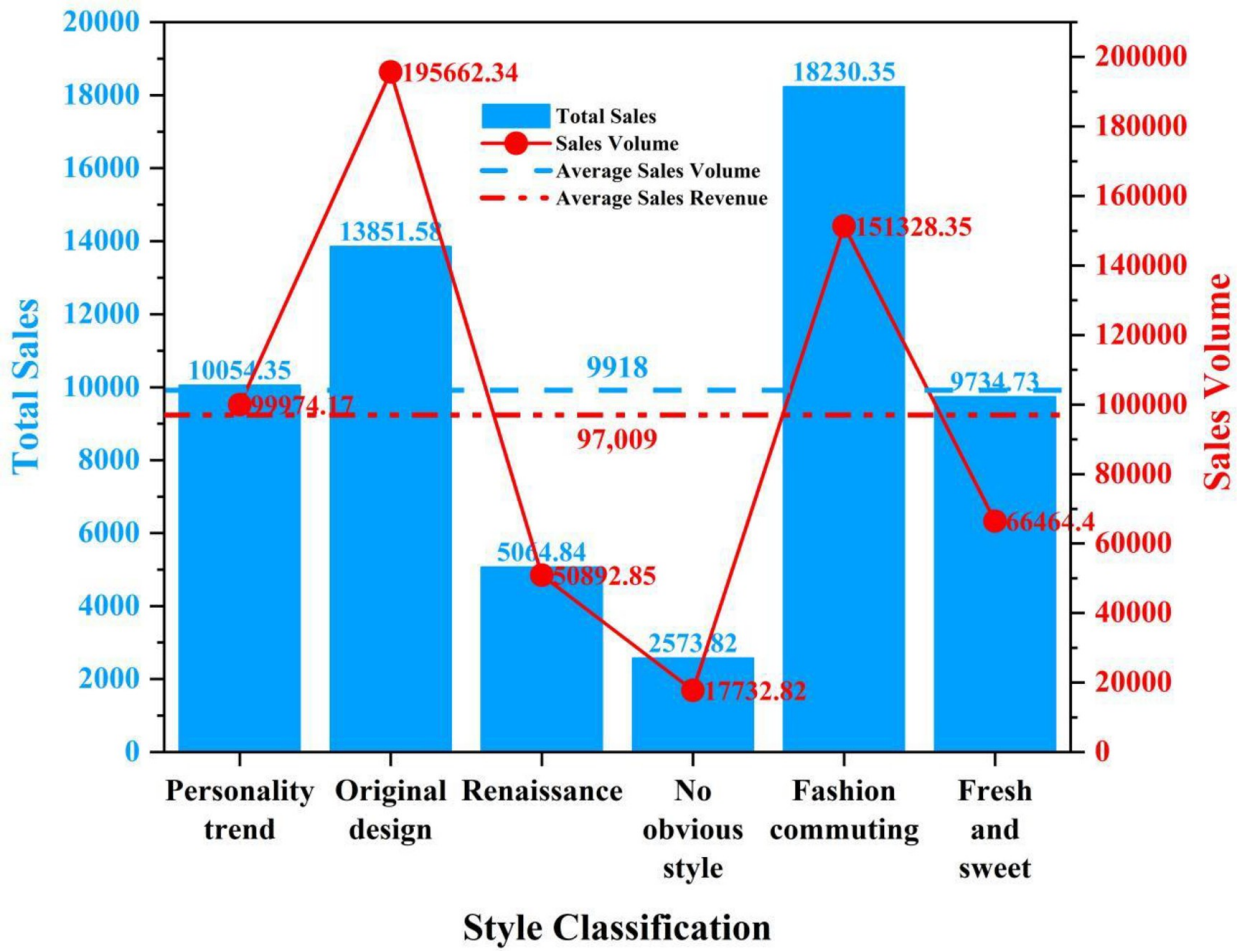
Straw hat style corresponds to sales and sales volume.

*(2) Popular elements and styles*. As a fashionable accessory, the style types and popular elements of straw hats are crucial to consumers. For merchants, keeping up with fashion trends is a vital pathway to increasing sales. Therefore, we conducted a word frequency analysis on the style and popular elements tags in the current straw hat sales data, resulting in the Fig 1:

The word cloud clearly illustrates the trend in straw hats, with "百搭"(versatile),"休闲风(casual)" and "简约"(simple) as the main directions, and styles like "欧美"(European-American) and "韩版韩式"(Korean) being more prevalent. This aligns with contemporary young people’s pursuit of comfort and a casual style. Additionally, we categorized the straw hat sales data by type and calculated the average sales volume and revenue for each category (see Fig 2). Hats tagged with "fashion commuting" achieved the highest average sales volume, followed by "original design." However, "original design" hats garnered higher average revenue, possibly because these hats include more design costs and brand effect, thus commanding higher prices. As expected, hats with "no obvious style" had significantly lower sales volumes and revenues compared to other tagged hats.

*(3) Customized design and applicable scenarios*. With societal progress and an increase in living standards, consumer demand has extended beyond basic functions to increasingly seek products that reflect their personality and life attitude. The diversification and personalization of consumer preferences are gradually driving reforms in merchant production strategies. Implementing product personalization not only meets consumers’ needs for uniqueness, creativity, and personal expression but also attracts a larger market.

**Fig 3:**
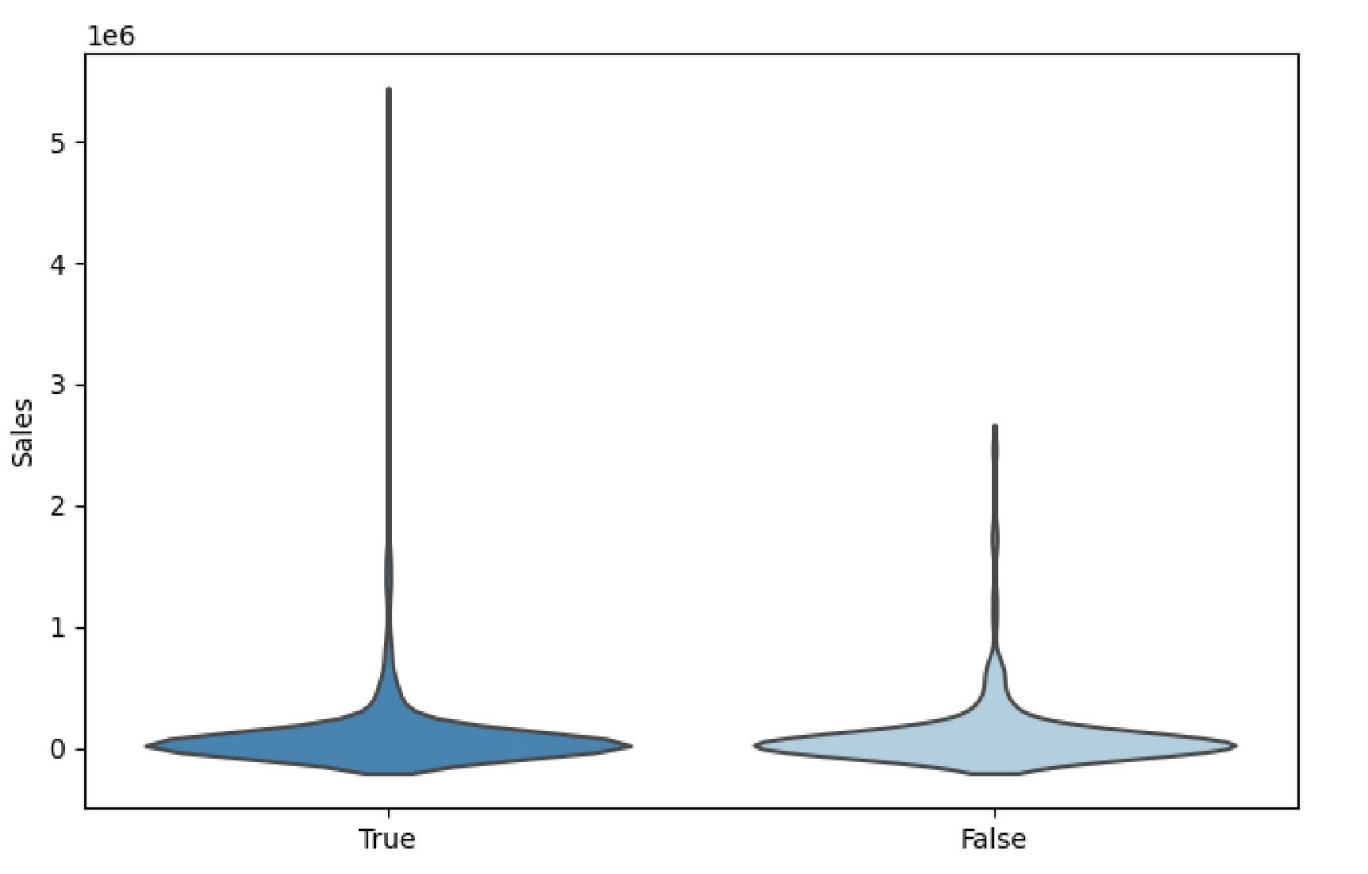
The relationship between straw hat sales and customization services.

On the data level, merchants offering customized straw hat services accounted for 72% of the total, including services such as "adding a logo,""processing according to provided designs,""adding rhinestones," and "camouflage." Merchants providing customization services achieved an average monthly sales revenue of 1198.68, whereas other merchants had an average monthly sales revenue of only 676.3, with little difference in average unit price between the two. However, given the significant disparity in the number of the former being more than double that of the latter, using the mean as a measure might not be the most appropriate due to the larger sample size’s potential significant impact on the mean. Therefore, we employed the median and interquartile range (IQR) to more accurately describe the difference in sales revenue between the two groups, as shown in Fig 3 and Table 3:

**Table 3:** The relationship between various statistics of straw hat sales and customized services.

Processing customization	Median	IQR	Number
True	16316.64	61266.6	489
False	13449.60	68572.8	193

From Fig 3, we can clearly observe a wider distribution in sales revenue among merchants offering customization services. This suggests that customized services have the potential to become best-sellers, thus achieving greater success in sales revenue. Moreover, observing the Interquartile Range (IQR) in Table 3, we find that merchants providing customized straw hats exhibit more stable profits in terms of sales revenue. This implies that the sales revenue of customized straw hats is less influenced by extreme values, presenting a relatively more reliable profit potential. Overall, customization services not only help to increase total sales but also reduce the risk of market downturn losses for straw hat sales, bringing more sustainable business returns to merchants. This has significant implications for formulating business strategies and product positioning.

**Fig 4:**
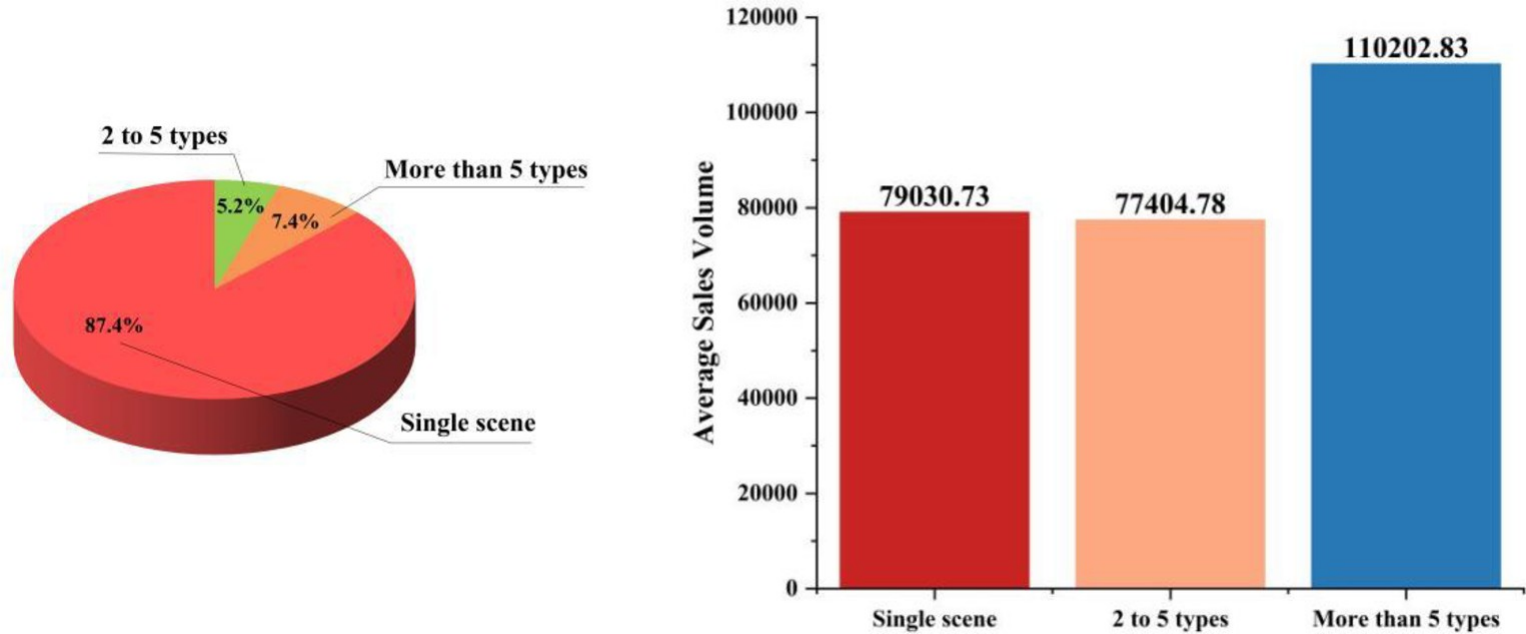
Schematic diagram of the applicable scenarios and sales volume of straw hats.

On the other hand, the popularity of versatile-style straw hats has drawn our attention, leading us to hypothesize that hats suitable for a variety of occasions might perform better in sales. To this end, we analyzed the relationship between the number of applicable scenarios for each product and its sales volume (see Fig 4).

Most straw hat products (87.4%) are limited to a single occasion, such as work, leisure, parties, etc. This indicates that straw hat production and sales are still dominated by assembly line mass production, focusing on the supply for specific scene demands. This often leads to some undesirable effects. On one hand, products suitable for a single scenario do not possess strong purchasing appeal in sales, leading to potential overstock and unsold inventory. On the other hand, in the search engines of e-commerce platforms, broader adaptability can significantly increase the probability of products being retrieved by keywords, thus attracting consumer attention and promoting online sales.

**Fig 5:**
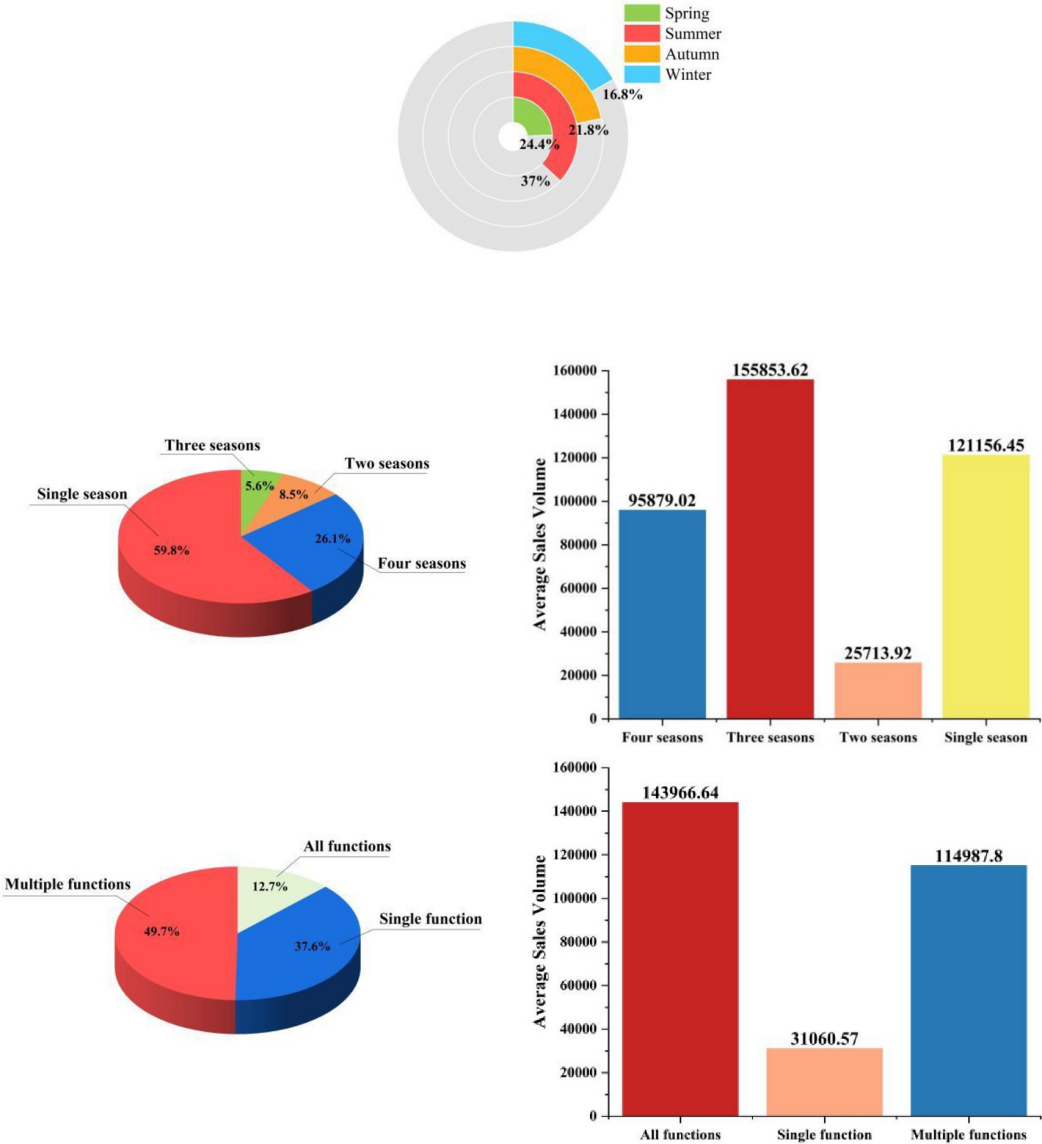
Schematic diagram of the season, function, and sales volume of straw hats.

The right-hand figure in Fig 4. intuitively validates our conjecture; hats suitable for more than five life scenarios achieve significantly higher average sales revenue. In contrast, hats suitable for a single scenario or a few scenarios have similar average sales revenues but are much lower than those adaptable to more than five scenarios. It is worth mentioning that we observed the same sales patterns concerning the applicable seasons of straw hats and their functions (see Fig 5): hats suitable for more seasons are favored by consumers; particularly, attributes suitable for winter tend to discourage purchases, as straw hats are perceived by consumers as products offering coolness and style rather than warmth. Regarding functionality, hats with more features undoubtedly attract consumers more, resulting in higher sales revenues.

From this, we can conclude that in the production of straw hat products, we should fully consider the multidimensional, multifaceted, and multi-scenario applicability of the product, especially highlighting this advantage on online platforms like e-commerce, which can more effectively attract a wide range of consumer groups. Furthermore, it is also necessary to pay close attention to the essential attributes of consumer goods, adding reasonable product attributes targetedly, as blindly pursuing versatility may lead to counterproductive effects.

#### 3.2.2 Consumer demand analysis

In the previous section, we systematically analyzed the impact of product attributes of straw hats on merchants’ revenues on e-commerce platforms and provided targeted suggestions. It’s important to note that these measures are applicable not only to the adjustment of domestic merchants’ sales strategies but also offer insights for cross-border business operations. Of course, to better serve the cross-border sales business, we need to delve deeper into overseas consumers’ demand for straw hat products. E-commerce platforms break down information barriers among consumers, containing a wealth of product reviews that can more accurately reflect consumer needs and product pros and cons. From this perspective, it’s essential to conduct an in-depth mining of customer review texts on cross-border platforms.

We chose Amazon (https://www.amazon.com/) as the data source. Amazon is the online retailer with the most extensive variety of goods globally and the second-largest internet company worldwide, covering users from almost all regions globally, meeting various overseas sales market needs. The platform also provides real, strictly reviewed buyer comments, which can be referenced by other consumers and, to a certain extent, reflect the advantages and disadvantages of the products. Therefore, we focused on customer review data of popular straw hats on the Amazon platform.

*(1) Price segmentation*. Unlike in China, the selling price of straw hats in the cross-border market is generally higher, mainly between $10 and $100. On the Amazon platform, straw hat prices are divided into three ranges: below $25, $25 to $50, and above $50; hats in different price ranges target different consumer groups, and their materials, designs, or brands vary significantly. For example, hats priced above $50 are often brand-name products, made of finer materials and usually handcrafted, so customer reviews may focus on aspects such as design and fashion compatibility. Meanwhile, for customers choosing hats priced below $25, practicality, wearing comfort, and the consistency between the actual product and its online presentation may be their concerns. Therefore, it is necessary to discuss straw hats in different price ranges separately, exploring their respective strengths or shortcomings.

**Fig 6:**
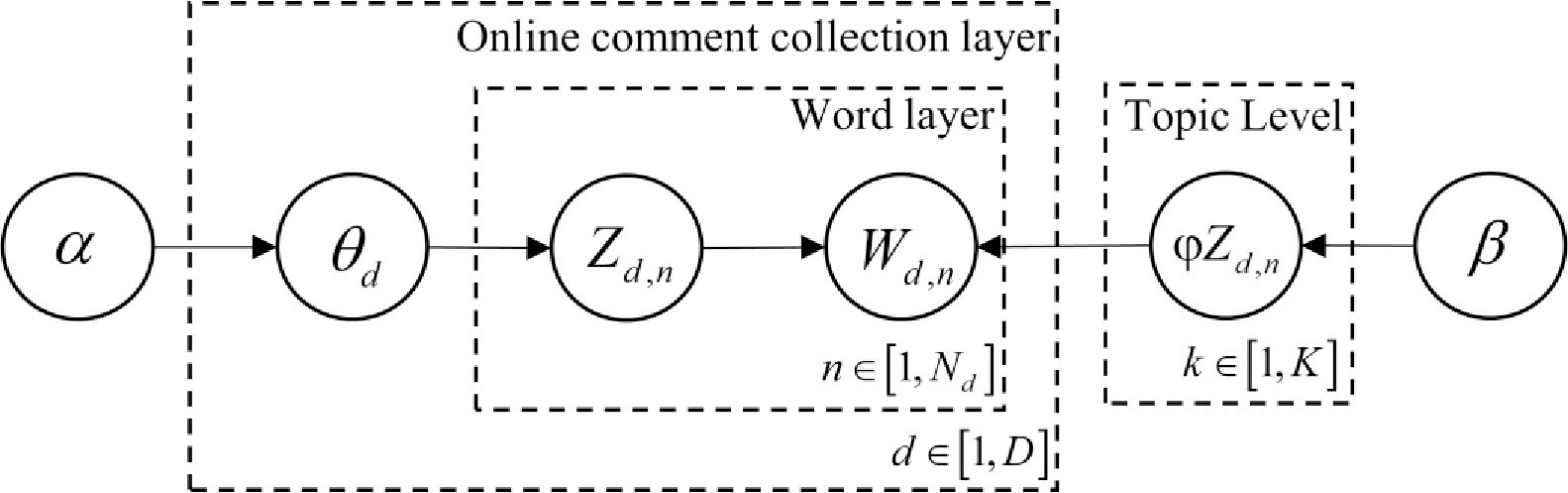
Cross-border e-commerce platform comment topic extraction model.

*(2) Straw hat consumer demand analysis based on the LDA topic model*. The LDA (Latent Dirichlet Allocation) topic model is a latent semantic mining model that explores the latent topic information in texts using an unsupervised learning method based on the Dirichlet distribution model. Essentially, the LDA model is a three-layer Bayesian probability model (see Fig 6), deriving the topics of an entire article by calculating the probability of word selection in the text. In simpler terms, LDA posits that a piece of text usually contains multiple topics, each corresponding to different words. It assumes that the words in a document are unorderly accumulated in a "bag of words." During a document’s generation process, each word in an article is considered to be obtained through a process where "an article selects a topic with a certain probability, and a word is chosen from this topic with a certain probability," as illustrated in the following process:

1. For each document in the dataset, the process involves extracting a topic from the topic distribution.
2. From the word distribution corresponding to the extracted topic, a word is then chosen.
3. This process is repeated until every word in the document has been traversed.

Formally, considering the entire collection of online reviews as the dataset *D*, where a particular review *d* is composed of *N* words represented as *w* = (*w*_1_,*w*_2_,…,*w*_*N*_) and assuming there are *K* latent topics within the collection of online reviews. The steps for extracting topics from the reviews using the LDA model can be summarized as:

1. Extract *N* words, with *N*~*Poinsson*(*ξ*).
2. Extract the distribution of topics in online reviews, with *θ*~*Dirichlet*(*α*).
3. For each word *W*_*n*_ among the *N* words, first determine its corresponding topic *z*_*n*_, with *z*_*n*_~*Multinomial*(*θ*); then, generate the corresponding word *w*_*n*_ based on the conditional probability *p*(*w*_*n*_|*z*_*n*_,*β*).

Here, *α* represents the Dirichlet prior parameter for the multinomial distribution of a topic under any given review, and *β* represents the Dirichlet prior parameter for the distribution of characteristic words under that topic; *θ* signifies the topic distribution across the entire review.

In the model, *K* represents the total number of topics, which can be predetermined; *D* represents the total number of reviews, *N*_*d*_ represents the number of words in the *d*−*th* review, and *W*_*d*,*n*_ represents the *n*−*th* word in the *d*−*th* review; *θ*_*d*_ and *φ*_*k*_ represent the topic distribution in the *d*−*th* review and the distribution of characteristic words under the *k*−*th* topic, respectively; *Z*_*d*,*n*_ represents the topic of the *n*−*th* word in the *d*−*th* review. It is important to note that *W*_*d*,*n*_ is an observable known variable, *α* and *β* are prior parameters estimated based on actual experience, and *Z*_*d*,*n*_, *θ*_*d*_, and *φ*_*k*_ are unknown variables, which can be estimated through learning from known variables.

In this paper, we employ the LDA model to mine the thematic elements within straw hat reviews across various price ranges, thereby identifying the needs of different target groups towards straw hats. For convenience, we utilize Python programming, specifically the LatentDirichletAllocation package from the Sklearn library, to implement an LDA-based model for extracting the online cross-border demand for straw hats. Initially, all reviews are cleansed to remove stop words that are irrelevant to topic representation, such as "just,""a,""the," etc. Subsequently, based on related literature, the total number of topics for the reviews is determined, with each topic comprising 5 words, and the word feature vector length set to 1000. The results are presented in Table 4:

**Table 4:** Online comment topics for each price range.

Price range	Theme	Content
Under $25	size	hat、fit、large、big、head. . .
	purpose	gift、travel. . .
	type	sombrero、curved、brim. . .
$25 to $50	service	picture、star、broke. . .
	occasion	windy、tie、job. . .
	color	white、ivory、colors. . .
Over $50	dressing	wear、looks、sun、nice. . .
	material	straw、plastic、material、coating. . .
	display	particularly、appearance、seam. . .

From the table, it’s evident that the focal points of straw hat evaluations vary significantly across different price ranges due to the diverse consumer groups they target. Analyzing consumer comments on lower-priced straw hats (under $25), it becomes apparent that consumers pay special attention to the hat’s size, practicality, and diversity. Common concerns include whether the straw hat fits well, whether it can adapt to different head shapes, and whether there is sufficient variety to meet needs for gifting or travel accessories. These insights reveal an important market trend: in the more affordable straw hat segment, consumers tend to focus on the product’s practicality and versatility. Based on these insights, it can be inferred that for low-priced straw hats, merchants should emphasize the product’s advantages in terms of size accuracy, style variety, and multipurpose use. By highlighting these features, merchants can better meet the needs of their target customer group and stand out in a competitive market. Thus, product descriptions, marketing activities, and product design should all be constructed around these key factors to attract price-sensitive consumers seeking value for money.

For mid-priced straw hats ($25 to $50), consumers’ purchasing considerations seem to lean more towards the consistency of the product’s online presentation and the overall shopping experience. Customers in this price range particularly value whether the product’s online presentation matches the actual item, as well as the overall reviews, customer service quality, and comprehensive after-sales support. This tendency indicates that consumer expectations for mid-range priced straw hats extend beyond the product itself to encompass all aspects of the purchase and post-purchase experience. Additionally, mid-range priced straw hats also exhibit a clear preference for functionality and applicability. Consumers generally expect these hats to be suitable for various scenarios, such as resisting strong winds, adapting to cloudy weather, and even being appropriate for workplace environments. This focus on versatility and situational adaptability reflects higher expectations from consumers for mid-range straw hats. Another important consideration is the hat’s appearance design, especially color choices. In this price range, solid-colored straw hats seem to be more popular, likely because they are easier to match with different outfits and occasions. Therefore, for mid-priced straw hats, merchants should consider offering a rich selection of colors to attract consumers with certain fashion and aesthetic demands. In summary, when developing marketing strategies for mid-range priced straw hats, merchants should focus on enhancing the online shopping experience, ensuring the accuracy and transparency of product information, while providing high-quality customer service and after-sales support. Additionally, product design should emphasize versatility and aesthetics, especially in color selection, to meet the diverse needs and aesthetic preferences of consumers. Through this comprehensive strategy, merchants can more effectively attract and satisfy the demands of the mid-range market.

Analyzing consumer comments on high-priced straw hats (above $50), we find that this group’s expectations align with the price, viewing straw hats as a fashion accessory and a work of art. These consumers care not only about how the straw hat complements their personal style but also value its unique design and craftsmanship. In other words, for these consumers, high-end straw hats transcend mere practical utility, becoming a means to express personal taste and lifestyle. Moreover, the consumer group for high-end straw hats places great importance on material selection and the artistic value of the hats. They tend to opt for products that not only showcase exquisite craftsmanship but also embody environmental principles. This indicates that for the high-end market, sustainability and the use of eco-friendly materials are not just value-adds but are key to meeting the growing environmental consciousness of this customer group. Therefore, marketing strategies for high-end straw hats should highlight their fashion elements and craftsmanship level. Product promotion should emphasize the artistry of unique designs and the sustainability of using eco-friendly materials. In this way, merchants can attract consumers seeking high quality and unique designs while also catering to those increasingly concerned with environmental protection and sustainable development. In the high-end market, by combining fashion with environmental responsibility, straw hats become more than just headwear; they represent a lifestyle and a set of values.

In conclusion, the straw hat market across different price points reveals varying consumer preferences and demands. Merchants need to adjust their product positioning and marketing strategies accordingly to better meet the needs of different consumer groups and stand out in a competitive market.

### 3.3 Formulating a sales strategy under the decision tree model

In our exploration of straw hat sales data and consumer reviews, we utilized various data analysis methods and algorithms to deeply mine the key factors driving straw hat sales as well as the needs and preferences of different consumer groups. Our analysis not only revealed the general demand trends in the straw hat market but also detailed consumers’ personalized needs. Sales data provided us with a macro perspective, reflecting the general popularity of straw hat products and market trends, revealing the common needs consumers have for different types of straw hats. Meanwhile, review data offered more microscopic insights, directly reflecting consumers’ real experiences and personalized preferences, enriching our understanding of consumers’ nuanced demands. With data from both aspects, we were able to construct a comprehensive and balanced market analysis framework. This not only enabled us to identify which factors most attract consumers but also understand the status and value of straw hats in different price ranges among consumers; and to devise a sales strategy that is both comprehensive and precise, aiming to meet consumers’ common needs while also paying attention to their individualized preferences.

In previous analyses, we explored various single attributes affecting straw hat sales, such as popular elements, customization services, color varieties, and applicable scenarios. Each of these factors significantly impacted straw hat product sales, but our analysis remained at evaluating each attribute separately. However, in reality, consumers’ purchasing decisions are often influenced by a combination of multiple factors, and the independent analysis of single attributes may not fully reveal the complete picture of what influences sales. Therefore, to more accurately devise product strategies for straw hats and optimize sales strategies, we need to further explore the combined effects of different attribute combinations. By analyzing the interactions and combined effects between attributes, we can identify which attribute combinations most attract consumers and drive sales growth. This multi-attribute comprehensive analysis will help us understand market dynamics and consumer preferences more deeply, thereby providing strong data support for formulating more efficient and targeted marketing strategies.

For this purpose, we adopted a decision tree model to predict the ideal straw hat sales attributes. The decision tree is a classic machine learning method used to predict the mapping relationship between data attributes and target values [41]; and as an important data classification technique, the decision tree can be trained through supervised learning to obtain a refined classification model. In this case, the decision tree can explicitly select straw hat attribute values to derive the optimal sales plan. For simplicity, we selected "straw hat style,""style,""suitable season,""color," and "service rating" as the data attributes to be trained, with "sales volume" as the target value of interest. Of course, we also need to preprocess the data as follows for better training:

1. Further cleanse the original data, removing null values and meaningless symbols; eliminate outliers to avoid severe training errors.
2. Discretize the attributes and target values. Since "color" attribute values are all categorizable strings, they need to be labeled for model training convenience. Knowing from the text that the number of colors available for straw hats can significantly impact sales levels, we consider grouping the "color" attribute into reasonable quantity intervals. According to the principle of equal frequency intervals, color quantities are named "very few,""few,""medium,""many," and "very many" for intervals of 1, 1 to 4, 4 to 6, 6 to 10, and more than 10 (interval inclusive on the left), respectively. Similarly, we can discretize the continuous "total sales volume" into "low sales" (0 to 24), "medium sales" (24 to 301), "high sales" (301 to 2689), and "very high sales" (2689 to 470939).

**Fig 7:**
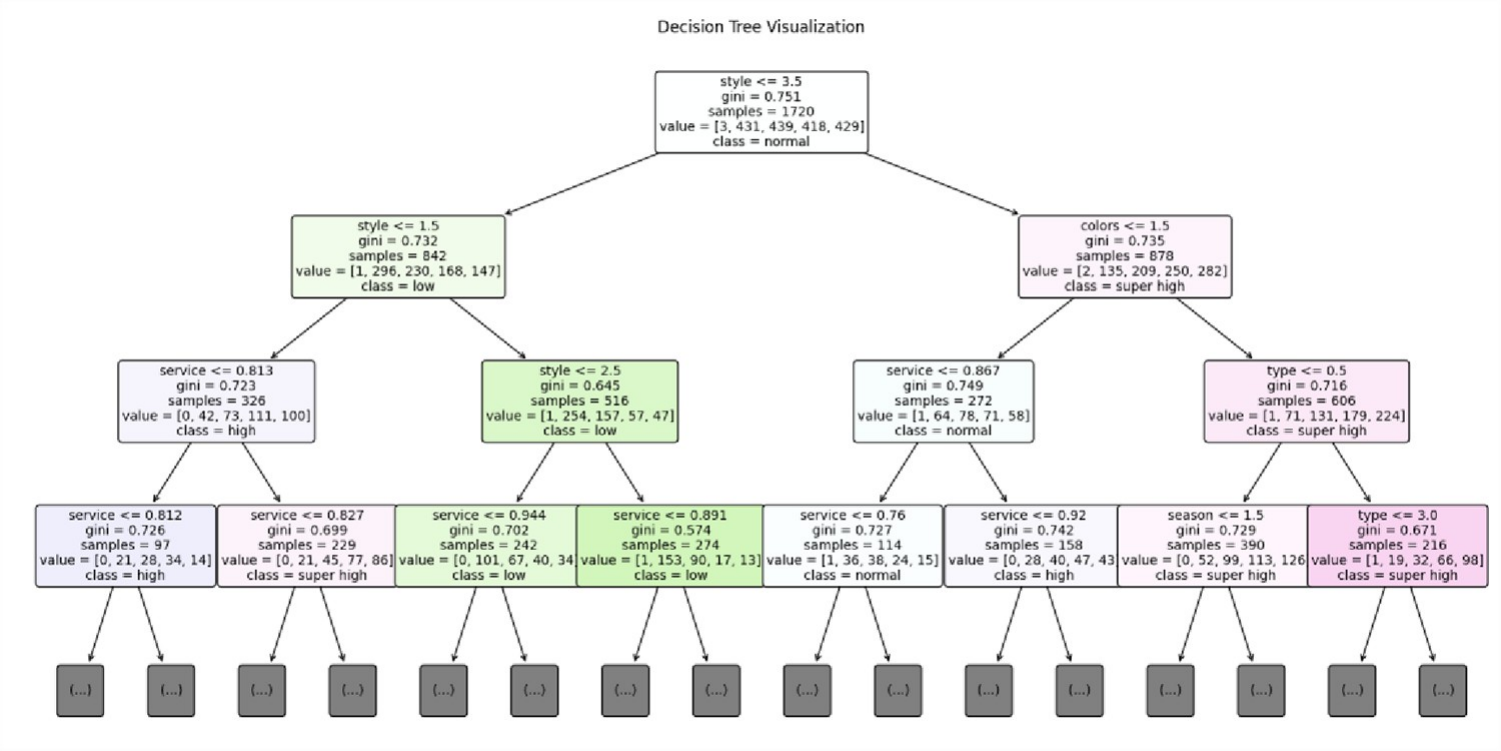
Visualization of straw hat attribute decision tree.

Ultimately, the data volume for decision tree analysis was reduced to 1812 entries, with the training and testing set split ratio set at 0.95:0.05, the results are detailed in Fig 7, Tables 5 and 6:

**Table 5:** Results of various indicators in the decision tree model.

	Accuracy	Recall	f1-score	Number of supports
Low sales volume	0.83	0.80	0.82	25
General sales volume	0.60	0.63	0.62	19
High sales volume	0.75	0.78	0.76	27
Extremely high sales volume	0.84	0.80	0.82	20
Average	0.755	0.753	0.755	

**Table 6:** The contribution of each attribute of straw hat to model classification.

Attribute Name	Contribution to the model
Service rating	0.5857
Color	0.1361
Style	0.1241
Straw hat style	0.0887
Suitable for the season	0.0654

Fig 7 showcases an intuitive visualization of the entire decision tree’s reasoning and classification process. For ease of presentation in the text, we have only captured the tree structure up to the first three layers. In this tree, each node represents a decision point for classification, through which the data is progressively refined and categorized. The Gini coefficient displayed in each node is an important indicator for assessing the quality of classification criteria [42]. The level of the Gini coefficient reflects the accuracy of the classification; a higher Gini coefficient means that the classification criteria based on that node can yield more accurate classification results. Table 5 displays the performance of the decision tree model on the test set; the model demonstrates excellent performance, specifically in terms of classification accuracy at 0.755, recall at 0.753, and an F1 score of 0.755. These results clearly indicate that the decision tree model is highly suitable for application in the attribute selection analysis for cross-border e-commerce of straw hats; this performance undoubtedly emphasizes its effectiveness and reliability in handling classification problems. Furthermore, we delved into the impact of various attributes on the model’s prediction outcomes (see Table 6). In this regard, the service rating of the merchant is evidently the most critical factor affecting consumers’ purchasing decisions, with its importance surpassing other attributes such as the color choices and style of the straw hats. In summary, dome-shaped and brimless straw hats are more popular among consumers, and the product style conforms to the current fashion mainstream with personalized trends, artistic retro, or original designs being preferred; the number of colors available for straw hats is best set at five or more, with spring and summer being the ideal seasons for these products. Beyond product attributes, merchants should also improve their operational level, optimizing the service level of the store to around 0.9.

In this section, we utilized the decision tree model to analyze and derive the optimal sales strategy for straw hat products. It’s worth mentioning that the model can incorporate more straw hat attributes for training, and the larger the volume of data, the more accurate the decision-making will be. On the other hand, sales strategies can change according to data variations, implying that the proposed model is capable of adapting to rapidly changing consumer markets and preference demands. By combining the analysis of different consumer target groups’ needs previously discussed, a more comprehensive and targeted strategic plan will be achieved, which will be reflected in the subsequent text.

## 4. AI-assisted industrial upgrade

**Fig 8:**
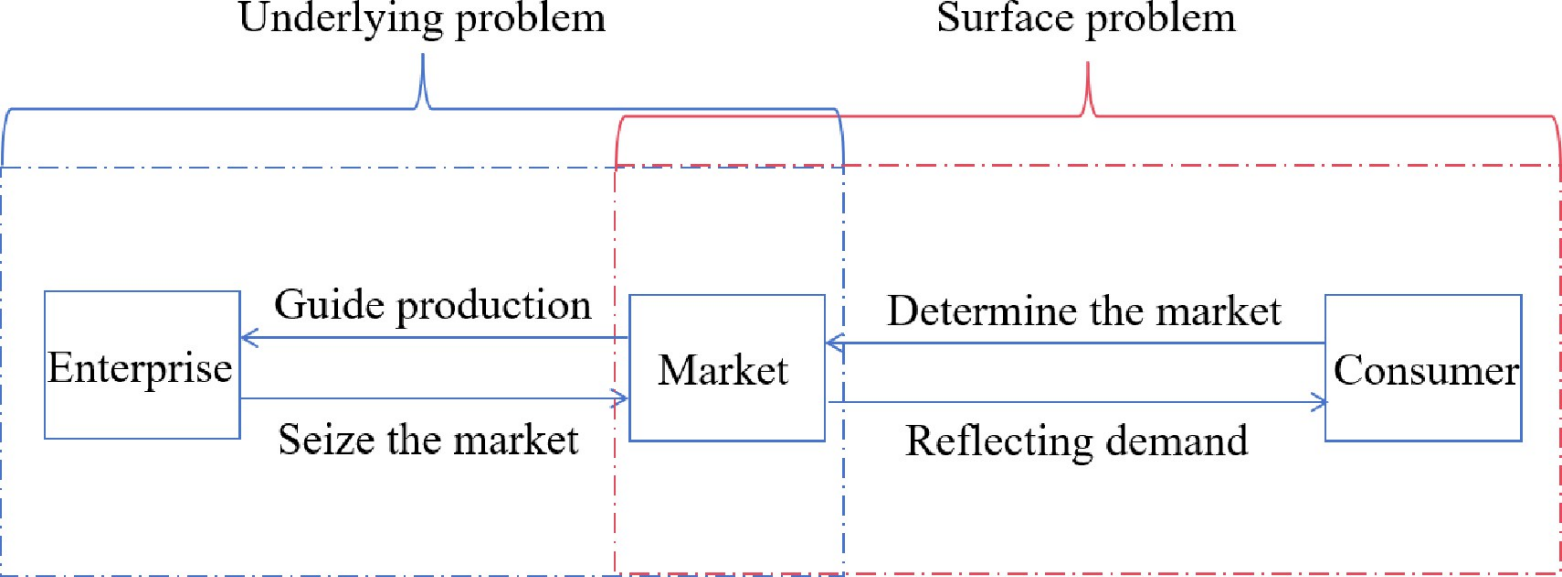
Schematic diagram of information exchange between enterprises, markets, and consumers.

The integration of big data analytics capabilities of e-commerce platforms with precise machine learning algorithms provides businesses with a more accurate and convenient means to discern market trends and consumer demands. This approach effectively addresses the communication and interaction issues between businesses and consumers, which we refer to as the "surface problem". It involves a deep analysis of consumer behavior and market dynamics. However, more critical is solving the so-called "underlying problem", which entails refining the management of external information to allow businesses to more effectively guide production strategies. This enables rapid capture of product hotspots and increase in market share, or through efficient and accurate marketing strategies, significantly enhance sales performance. Therefore, we must pay more attention to improving the market response speed and production efficiency of enterprises (see Fig 8).

### 4.1 Traditional cross-border trade order processing

**Fig 9:**
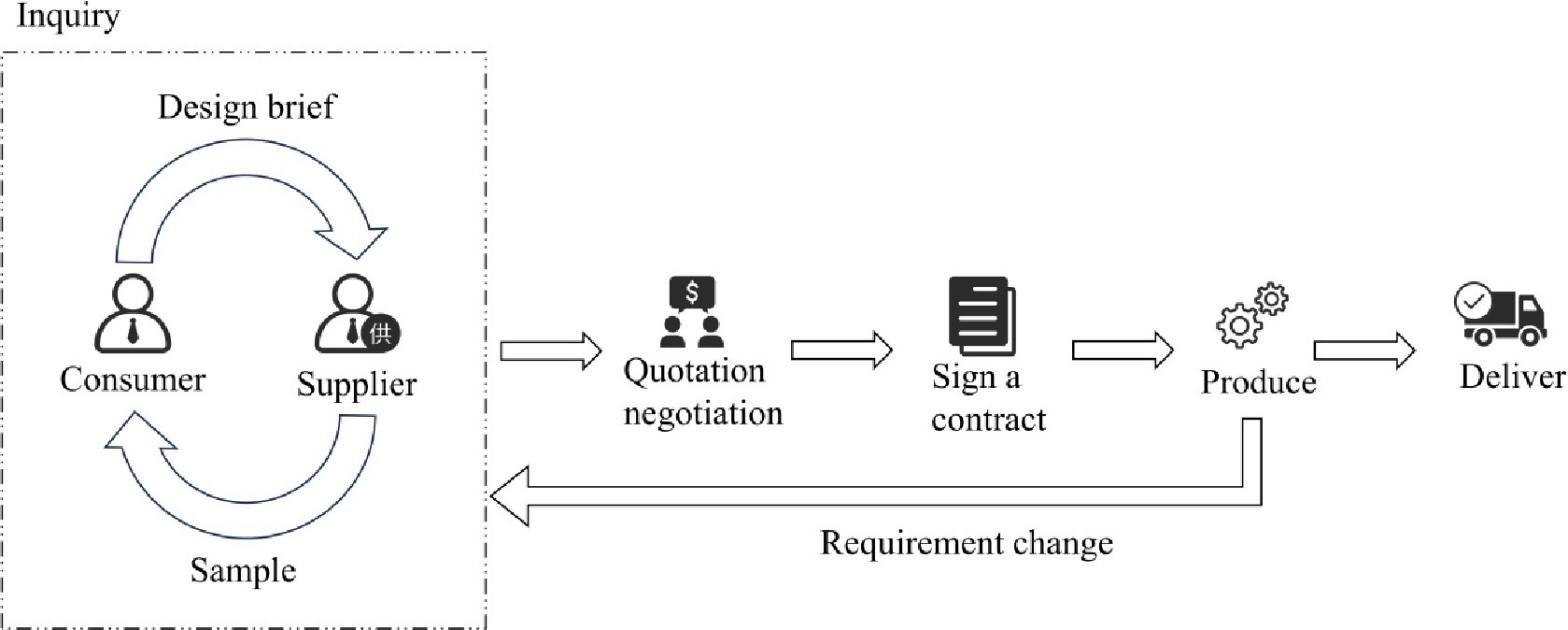
Traditional foreign trade order processing flowchart.

In the traditional cross-border trade order processing flow, the following steps are typically involved (see Fig 9):

(1) Inquiry Phase: Customers send inquiries to suppliers, asking for information on product prices, delivery times, quality standards, etc. For transactions involving apparel items like straw hats, the buyer may further provide product design requirements, such as design outlines, sizes, colors, materials, etc., which are very important details in the order contract.

(2) Quotation and Negotiation: Based on the customer’s requirements, suppliers provide a rough quotation for the products and negotiate on price, delivery conditions, payment methods, and other aspects. During this stage, suppliers usually need to produce samples after receiving the customer’s product requirements for the customer’s review to ensure both parties have a clear and precise understanding of the product design. This reduces errors in the later stages and facilitates contract signing.

(3) Contract Signing and Production: Once the terms of cooperation are agreed upon, a formal contract is signed. The supplier begins production preparation and develops a production process according to contract requirements and customer needs, ensuring the produced goods meet the agreed quality standards. It is noteworthy that if the customer’s requirements change, it might be necessary to return to the previous stage.

(4) Delivery and Settlement: The supplier delivers the products on time and in the required quantity. The customer pays the corresponding fees according to the contract, thus settling the order. The supplier also needs to provide after-sales service, handle customer feedback, resolve issues, and establish long-term cooperative relationships. Additionally, it’s beneficial to record and analyze order processing, summarize lessons learned, optimize processes, preserve production data, and improve the efficiency and quality of future order processing.

From the aforementioned process, especially in cases where customers and suppliers are geographically distant and primarily rely on online communication, a significant amount of time often needs to be invested in discussing and understanding product requirements [43]. Through research conducted on several straw hat manufacturing companies, it has been found that the inquiry phase is the most critical part of the order processing. In this stage, manufacturing companies need to create sample previews based on design drawings or sketches provided by the customer, with the involvement of designers and graphic artists. However, this is just pre-production preparation. As the production process advances, focusing on customization demands requires adjustments to production plans and processes according to specific custom requirements, often leading to repeated communications between customers and suppliers. This, in turn, significantly increases time and labor costs. It is estimated that these costs could account for 30% to 60% of the total order processing costs, presenting an urgent issue that needs to be addressed.

### 4.2 AI-assisted cross-border trade order processing

**Fig 10:**
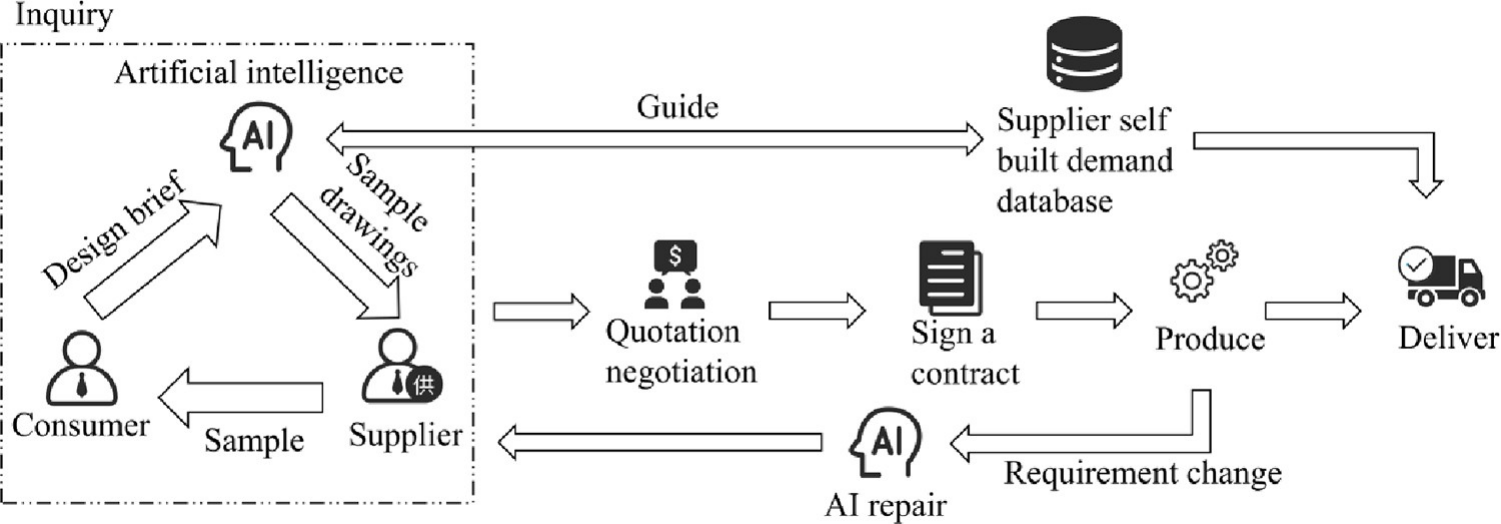
AI empowered foreign trade order processing flowchart.

To tackle this challenge, our focus shifts towards enhancing the efficiency of demand matching between customers and suppliers, aiming to reduce costs and improve overall efficacy. A viable strategy involves leveraging Artificial Intelligence (AI) technology to replace repetitive manual labor [44–46]. By integrating AI, we propose a new order processing flow designed to streamline existing steps, minimize unnecessary time consumption, and significantly boost efficiency (see Fig 10):

A comparison between Figs 9 and 10 clearly illustrates the improvements made to the entire production processing flow:

1. Suppliers no longer need to directly interface with customers for demand matching, as AI takes over the intermediary processes. This means customers are not required to provide highly precise design briefs; AI automatically understands and generates the corresponding sample drawings for the supplier, thereby accelerating the sample production and demand verification process.
2. Mid-production changes in product requirements can be responded to more swiftly. In traditional order processing, a seemingly simple request from the customer (such as changing the color) could lead to significant rework for designers and updates to the renderings, consuming considerable human and material resources. Through AI, the presentation of new requirements can be significantly expedited.
3. Suppliers create their own order databases. From our real-world enterprise visits, we learned that over years of product sales, companies accumulate a vast array of customer demands and corresponding product drawings. This indicates that new order demands can be quickly matched with suitable sample drawings from a large historical order database, thus saving a significant amount of time and cost.

### 4.3 Technological implementation pathway

#### 4.3.1 Generative AI technology

The year 2023 marks a period of rapid advancement in Artificial Intelligence Generated Content (AIGC) technology, with a variety of models emerging quickly and shining brightly. GAI stands for "Generative Artificial Intelligence," representing a type of technology that uses advanced AI techniques such as Generative Adversarial Networks and large pre-trained models to analyze and learn from existing data, possessing the ability to generate relevant content [47]. The essence of this technology lies in the deep learning of vast amounts of data, allowing AI to create content according to given conditions or instructions, seemingly endowed with human-like creativity. A notable example is ChatGPT, which has attracted considerable attention and controversy since the second half of 2022, capable of providing reasonable answers to user queries.

**Fig 11:**
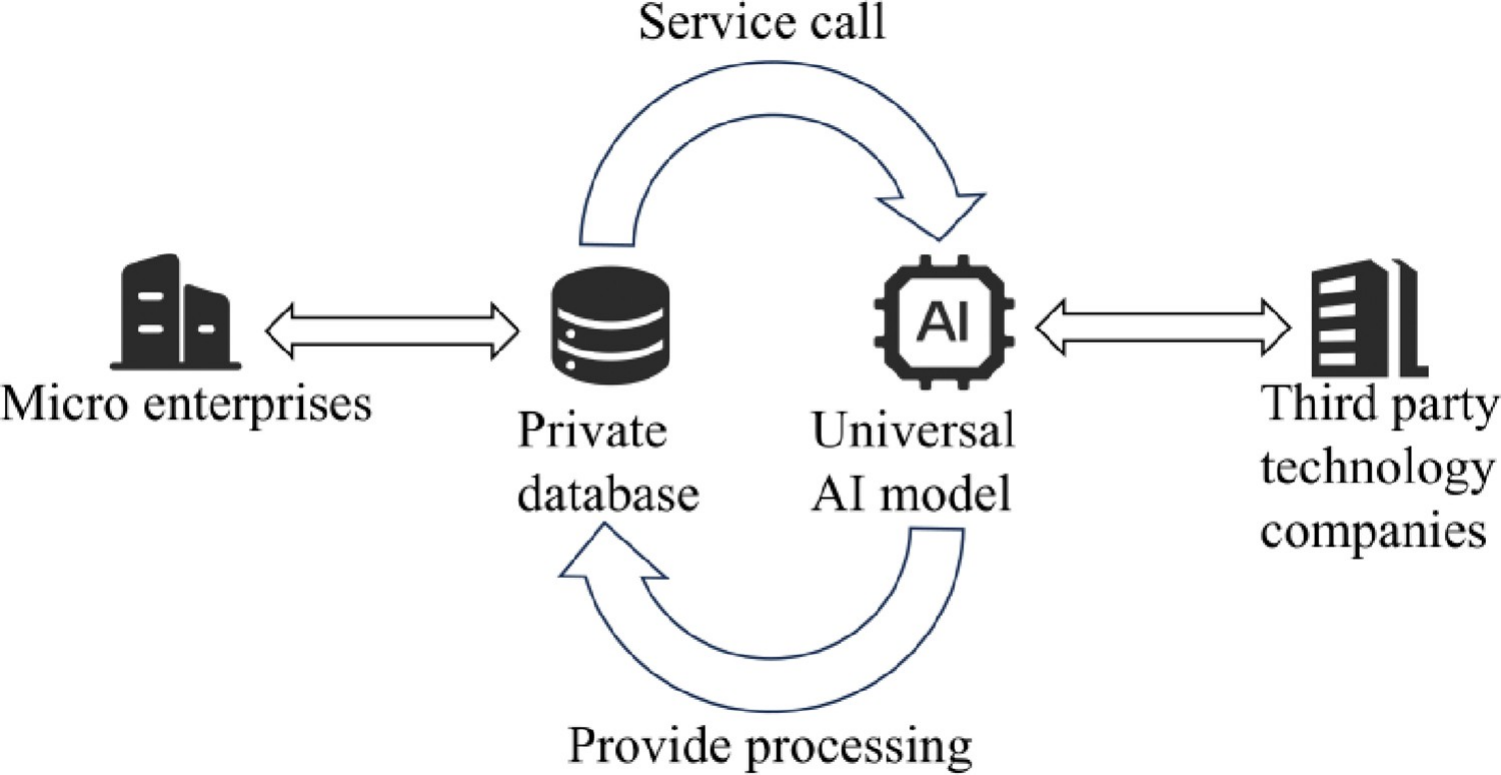
Schematic diagram of online use of AI models for micro enterprises.

However, the application of AIGC technology is not limited to the creation of simple text content. With continuous technological advancements, modern AI can now excel in image, voice, and even three-dimensional modeling. For instance, in image generation, users only need to provide a simple description for AI to produce highly matching images. This significantly enhances production efficiency and is expected to bring unprecedented convenience and business value to various industries in the foreseeable future [48]. Yet, this new business perspective also brings forth a variety of challenges: firstly, high-quality AI models typically require a vast amount of training data and substantial costs [49], which may pose a problem for small and micro enterprises. A common solution is for specialized third-party tech companies to provide AI services to these businesses (as shown in Fig 11), though the cost of such services can be quite high. Hence, balancing the cost of these services against the benefits they bring remains an unresolved issue. Secondly, reliance on third-party services could expose sensitive corporate data to leakage risks, necessitating strict control over model security. Moreover, third parties usually offer generic AI models, meaning that customized functionalities tailored to specific industry needs await further development and refinement.

#### 4.3.2 AI drawing technology for straw hat enterprises

**Fig 12:**
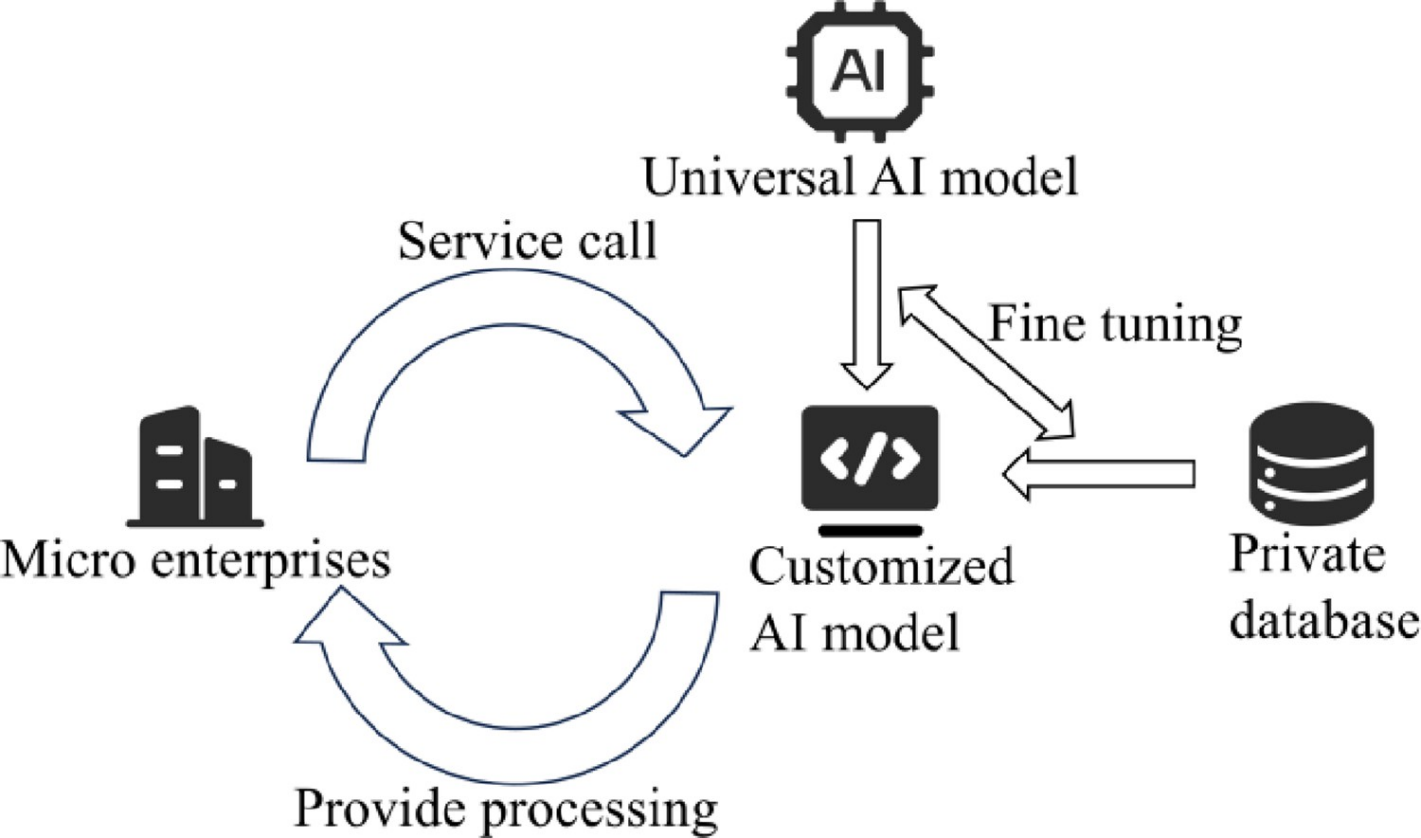
Schematic diagram of using private AI models for micro enterprises.

From the analysis above, we can conclude that while existing AI models have reached a mature, commercially viable stage, several improvements are needed to better serve straw hat enterprises in this report (as shown in Fig 12): 1) Isolate model usage, transitioning from online model service calls to local deployment. 2) Model self-training, customizing model training on the enterprise’s own database. The former approach, through local setup of AI models, avoids the exposure of corporate private data, ensuring data security; the latter adopts additional training strategies, fine-tuning model parameters to tailor-make suitable AI models for the enterprise, better adapting to business needs and order processing.

### 4.4 The case of Cixi Jusheng Straw Hats

To demonstrate the realism and effectiveness of the proposed architectural system, we have selected Jusheng Straw Hats, a local straw hat manufacturing enterprise in Cixi City, Zhejiang Province, as the benchmark company for this report and will discuss the corresponding improvement effects.

#### 4.4.1 Selection of a general AI model

The leading AI drawing models internationally include Stability AI’s Stable Diffusion, OpenAI’s DALL-E, and Midjourney. Within China, major AI drawing models are developed by seasoned internet technology companies, such as Baidu’s Wenxin and Alibaba’s Luban (details are shown in Table 7). As these models target different markets and development purposes, each possesses unique advantages. For example, DALL-E is known for its exceptional creativity and complex language understanding capabilities, making it highly suitable for users pursuing unique artistic creations. Midjourney exhibits more abstract and personalized creative abilities, ideal for film scene design and game development. Meanwhile, Baidu’s Wenxin and Alibaba’s Luban excel in understanding Chinese text, making them particularly suitable for illustration creation, advertising creativity, and layout design, offering a high cost-performance ratio. Moreover, most models are not open-source to the public, and their functions and services are usually limited to post-application calls.

**Table 7:** Comparison table of information for various AI models.

model	source	company	advantage	inferiority	Open source or not
Stable Diffusion	abroad	Stability AI	Rapidly generate high-quality, lifelike images	May not fully meet user expectations	Open Source
DALL-E	abroad	OpenAI	Powerful creativity, capable of handling complex text descriptions	The generation process is slower and may deviate from specific requirements	Partial open source
Midjourney	abroad	Midjourney	Abstract personalized creative ability, suitable for film and game design	The generated images may be overly artistic, not suitable for all purposes	Non open source
Baidu Wenxin	domestic	Baidu	Understanding of Chinese text, suitable for illustrations and advertising creativity	May not be as effective as models specifically designed for other languages	Non open source
Luban	domestic	Alibaba	Powerful design and layout capabilities, suitable for commercial promotion	More focused on design and layout rather than image creation	Non open source

Considering the cost pressures small and micro enterprises face during operation, choosing a free, open-source AI model naturally becomes our priority. Additionally, given that our services are primarily aimed at overseas markets, AI models developed within China might not fully meet our expectations; hence, we consider adopting a model developed internationally. A particularly important consideration is ensuring the company’s data security; we plan to conduct offline training of the chosen model, effectively avoiding risks of data leakage or loss. Taking all these factors into account, We decide to use the open-source general model, Stable Diffusion, as our foundational framework. It can be downloaded from the public link: https://github.com/Stability-AI/stablediffusion. This choice not only considers cost-effectiveness but also balances technical performance and application flexibility, providing us with an economical and efficient solution.

#### 4.4.2 Training the AI model based on historical order data

The general-purpose AI model mentioned in this report is a concept specifically for the AI drawing domain, which refers to a model capable of adapting to nearly all types of image generation tasks after extensive learning and analysis of a wide range of image types. In essence, the hallmark of such models is their broad generative capability, rather than a deep specialization in a particular niche. This presents two issues in the existing technological approach; on one hand, since general models typically use broad, unverified training datasets, this potentially raises the risk of image copyright infringement. Even if the images generated by the model are entirely new, the training process involves data that may contain copyrighted images, thus introducing a degree of legal uncertainty on copyright issues. On the other hand, although these general models can generate a variety of images, they often lack in texture details and in reflecting a company’s unique style. During the inquiry phase, customers tend to focus on the detail and personalized features of images, which is something general models struggle to fully satisfy. Therefore, training a customized AI model for the enterprise becomes particularly important in this scenario. Using the company’s own data for specialized training not only avoids copyright issues but also ensures that the generated images more closely align with the enterprise’s specific needs and style. This customized approach helps create higher quality and more personalized images, better meeting the specific needs of the business and its customers.

During a preliminary visit to Jusheng Straw Hats, it was discovered that the company is not only the largest local producer and exporter of straw hats but has also accumulated a wealth of overseas orders and customer demand information. This valuable data not only provides the company with strong customization service capabilities but also supplies ample resources for training the AI model. On one hand, these rich datasets ensure the effectiveness of AI model training; on the other hand, Jusheng Straw Hats has continually innovated over the years, successfully integrating traditional culture and green environmental concepts into product design, and has a precise grasp of foreign consumer needs, offering invaluable guidance for AI model training.

**Fig 13:**
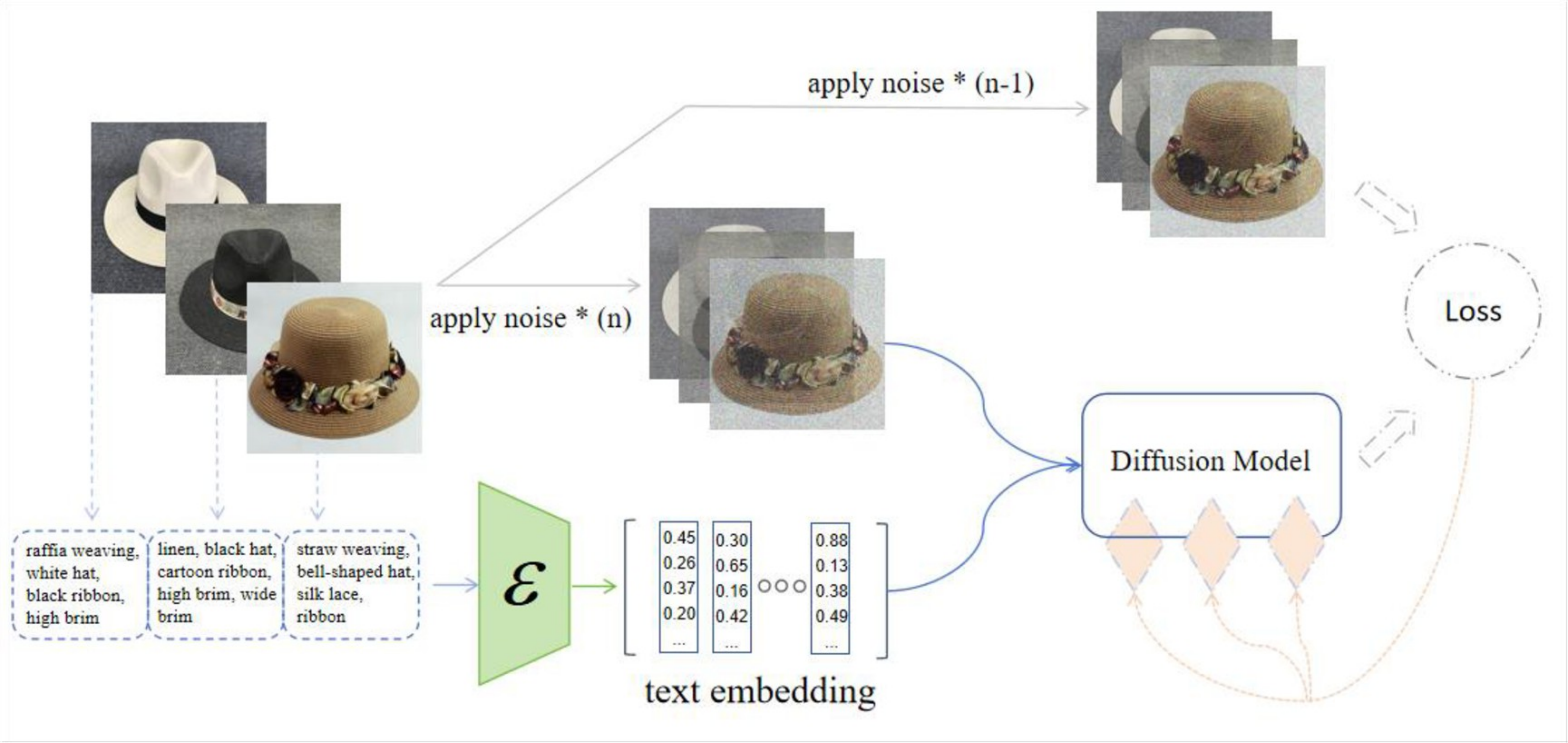
Training diagram of enterprise private model based on historical order data.

Additional training based on proprietary corporate data does not require the general model to be trained from scratch, avoiding the massive overhead of hardware and computational support. We adopted a "low-resource fine-tuning" approach [50], reducing the training parameters to one ten-thousandth of the original volume, cutting down on two-thirds of the device’s memory overhead without introducing additional inference time. The training data consisted of text pairs of order requirements and final sample images, trained in accordance with Low-Rank Adaptation methods, as illustrated in Fig 13. It consists of a pre-trained language encoder and a base diffusion model with fine-tuned training on a private data set. We followed the work from https://github.com/microsoft/LoRA to adjust the size and format of the images for better training, it’s worth noting here that the data used for training in this report all come from the enterprise, totaling about 3,000 entries, ensuring sufficient model training and fitting.

#### 4.4.3 Model application and effect demonstration

**Fig 14:**
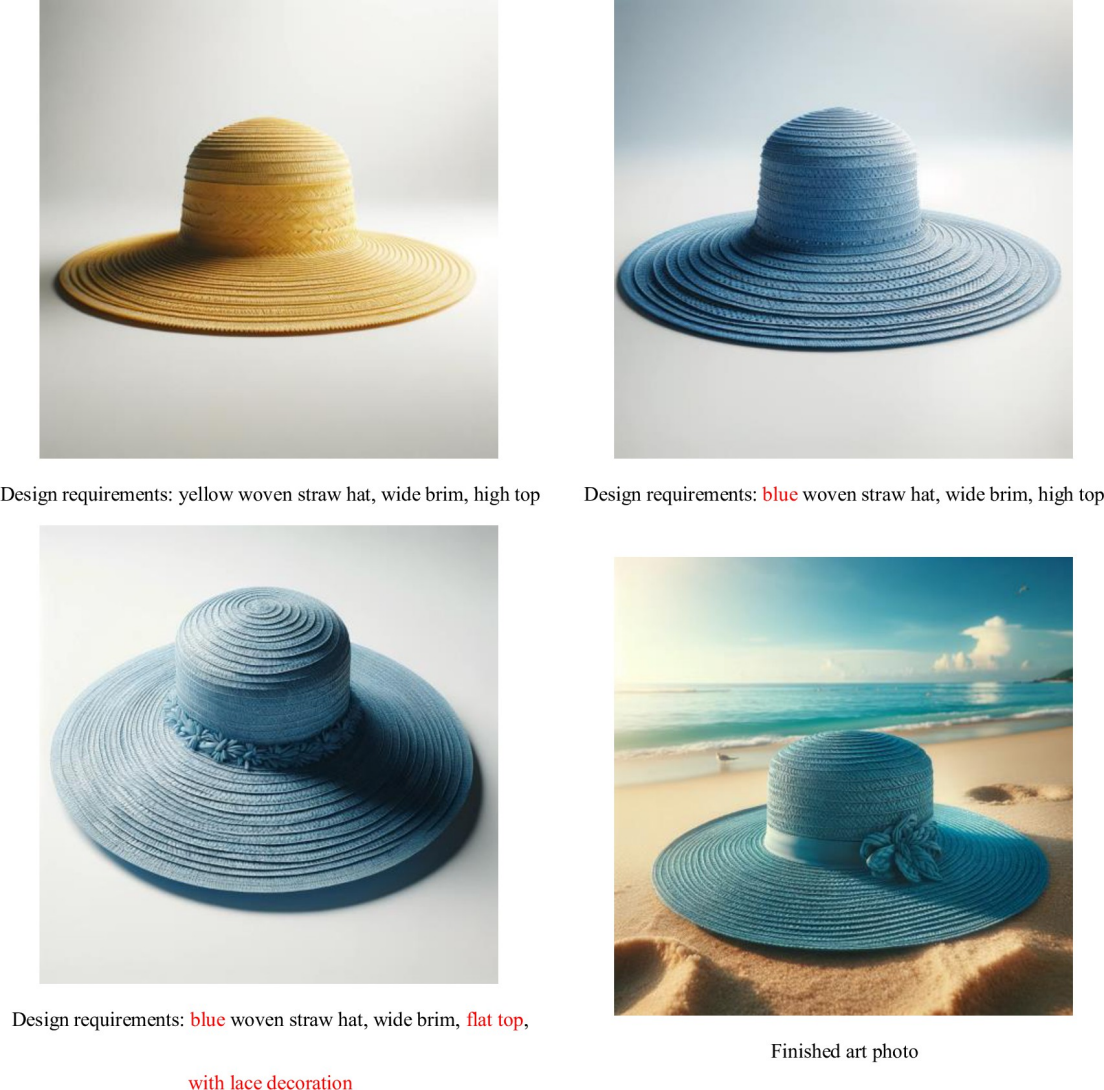
Generate a design sketch display diagram for order requirements, with changes to the requirements highlighted in red.

**Fig 15:**
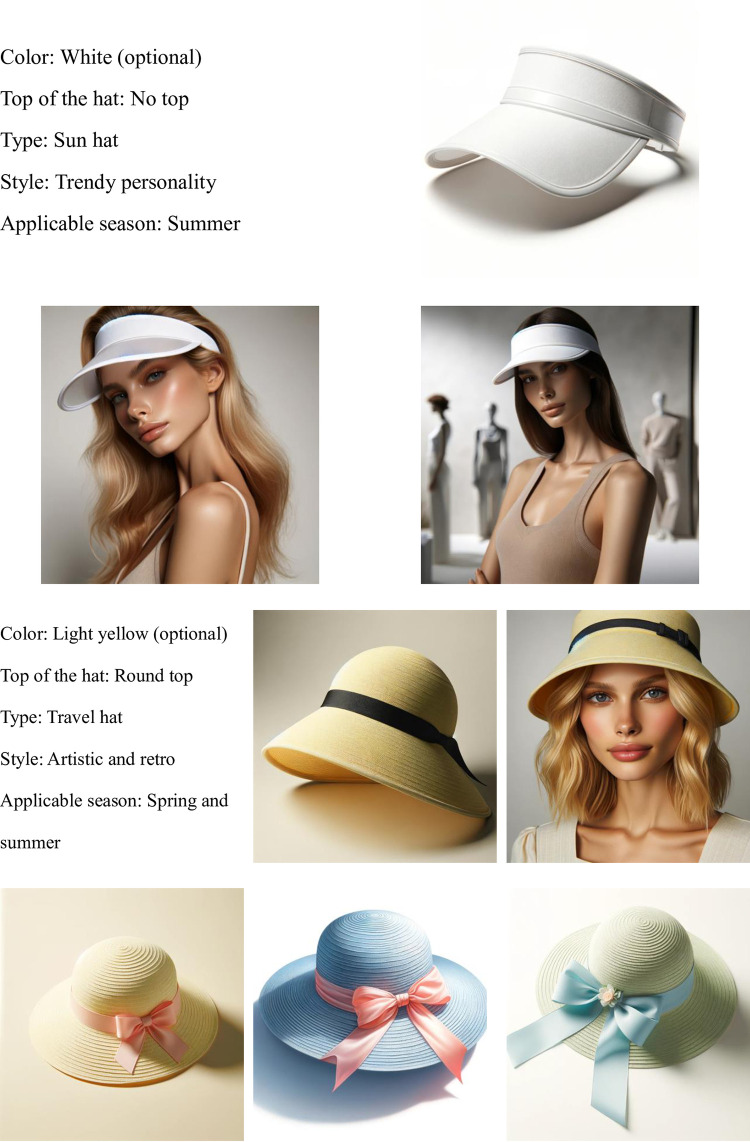
Visualization of high sales straw hat attributes.

**Fig 16:**
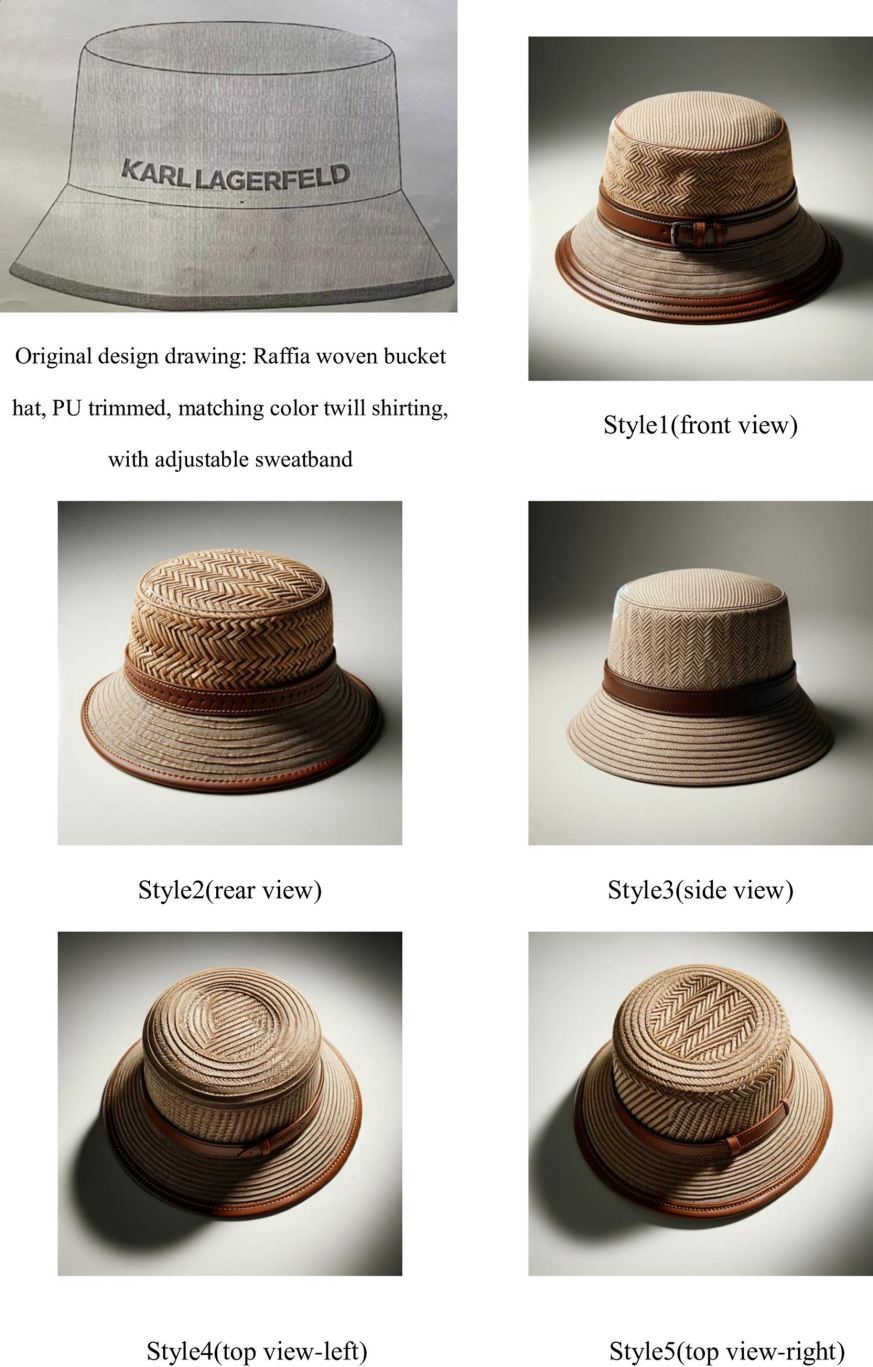
Design sketch refinement schematic diagram.

To verify the feasibility and effectiveness of our solution, we now present a demonstration of the model’s effects, as shown in the Figs 14–16:

It is evident that our AI model not only has the capability to generate high-quality sketches suitable for sample production but also ensures that the texture details of the images are presented more finely and vividly. It can accommodate various changes in demand, such as adjustments in color and style, simplifying what would typically be a time-consuming modification process to almost no waiting time. This is all thanks to our unique training dataset, which not only ensures that the generated images’ style remains consistent with the company’s production standards but also captures the brand’s unique charm and detailed features, thereby enhancing brand recognition. Moreover, our AI model supports the generation from requirements (in text form) to finished products (in image form) and can further refine design sketches, that is, generating refined sketches (in image form) from rough sketches (in image form). This significantly simplifies pre-production labor and time costs and allows the integration of traditional intangible cultural heritage weaving techniques into the products (different styles correspond to different weaving techniques, see Fig 16).

Furthermore, the high customizability and flexibility of the model mean it can easily adapt to changing market demands and innovation trends, opening new creative spaces and market opportunities for enterprises. Through deep learning and artificial intelligence technology, our model not only enhances design efficiency and productivity but also significantly reduces error rates and costs by automating complex design processes.

## 5. Marketing strategy framework

In this paper, we have detailed the use of machine learning algorithms and data analysis techniques to identify sales data on e-commerce platforms, thereby determining which attributes play a crucial role in the success of best-selling straw hat products. Text analysis techniques such as LDA can further enable businesses to extract valuable opinions and preferences from massive consumer feedback, thus better understanding market demands. Moreover, with the aid of AI, these key attributes and demands identified through analysis can be rapidly transformed into specific product designs (see Fig 15). This technology not only significantly shortens the cycle from design to production but also allows businesses to quickly adjust product designs based on real-time market feedback, achieving true on-demand production.

**Fig 17:**
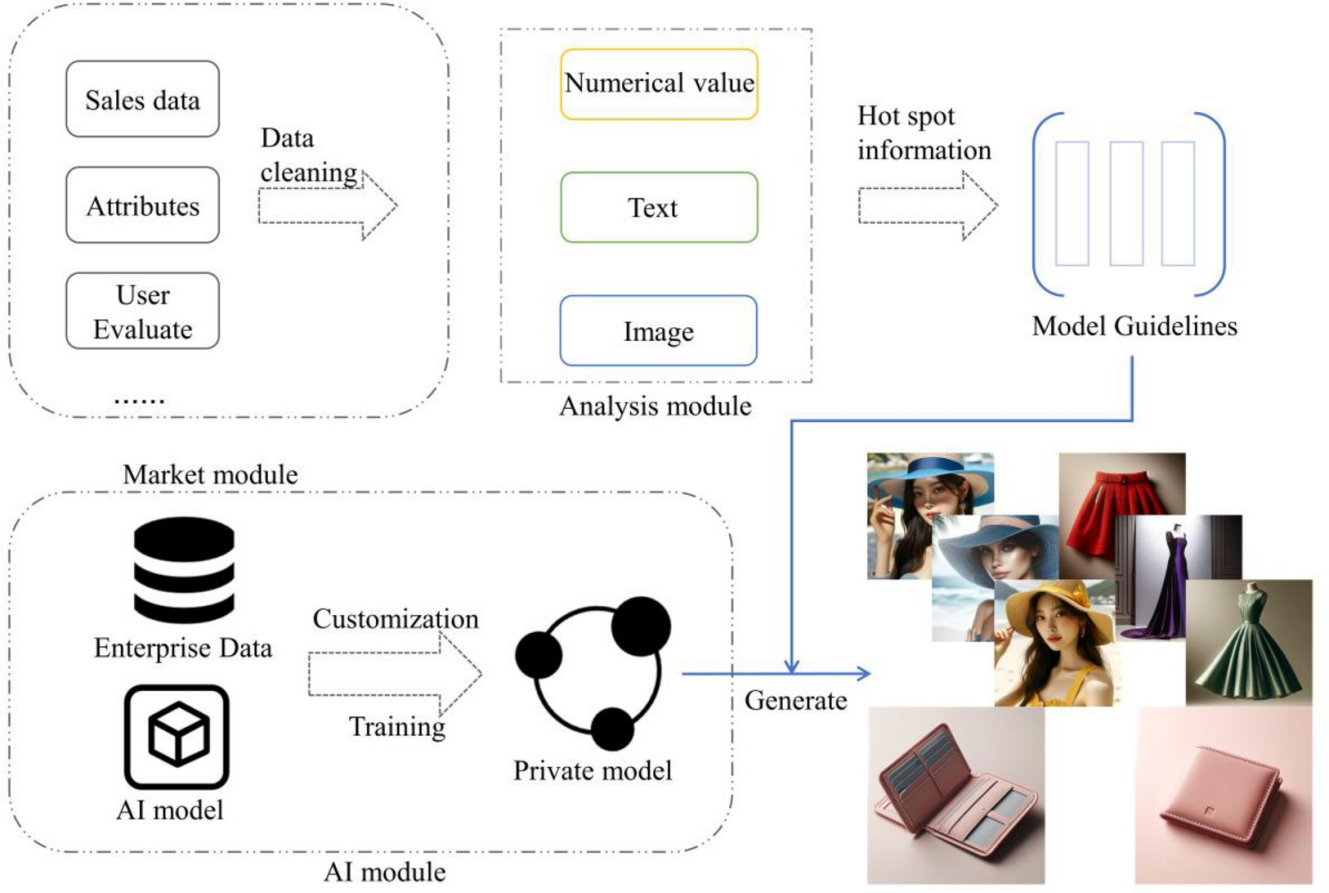
Integrated and pluggable marketing framework.

Therefore, we have constructed a marketing strategy framework based on big data, machine learning, and AI, focusing on three key components: market demand analysis, comprehensive product R&D, and adaptation to downstream tasks. By integrating precise machine learning algorithms with advanced AI models, this framework aims to optimize the straw hat industry’s process comprehensively, including market analysis, product positioning, and production marketing, among other crucial aspects (see Fig 17). This strategy not only enhances industry efficiency but also ensures products are more aligned with market needs, while accelerating the R&D and launch speed of products, achieving a comprehensive upgrade and innovation of the industry.

The Market Module is primarily responsible for aggregating data streams from various e-commerce platforms, including but not limited to real-time sales data, product attributes, and user reviews. After preliminary cleaning and processing, the data is efficiently distributed to specific processing units to accurately extract the optimal parameters of product attributes and the specific demands of the target consumer group. Simultaneously, another aspect of the framework focuses on utilizing the proprietary data of the target enterprise to provide customized production services. The main process involves receiving high-value product keywords and detailed preference analysis results generated after algorithm analysis. This data is then fed into a pre-trained enterprise-specific model to produce corresponding production sketches and marketing copy. It is worth emphasizing that our framework adopts an end-to-end integrated design philosophy, capable of dynamically adjusting production in response to immediate market changes to maximize benefits. This approach also allows for highlighting products’ hot attributes in subsequent actual platform sales for precise marketing; this aspect is also a significant manifestation of the framework’s plug-and-play properties.

## 6. Conclusion

This research paper has delved into the transformative effects of artificial intelligence (AI) and big data analytics on optimizing cross-border e-commerce efficiency, particularly for straw hat manufacturers in Zhejiang Province, China. Through comprehensive data analysis and the application of machine learning algorithms, our study uncovered significant insights into market and consumer demand trends, which have been pivotal in revolutionizing production and marketing processes for these enterprises.

Our findings underscore the remarkable potential of AI and big data in enhancing market responsiveness and sales performance in the straw hat industry. The integration of AI-generated content (AIGC) technology has not only streamlined the design-to-production cycles but also facilitated rapid adaptation to market changes and consumer feedback, thereby bolstering the competitive edge of small and micro enterprises in the global market. Furthermore, the research has contributed a novel framework for leveraging AI and big data in navigating the complexities of international commerce. This framework is not just a testament to the power of technological innovation in transforming traditional industries but also serves as a strategic guide for other sectors seeking to harness the benefits of AI and big data analytics for cross-border e-commerce endeavors.

In light of the challenges and opportunities identified, future research should continue to explore the evolving dynamics of AI and big data in e-commerce, with a focus on developing more sophisticated models and algorithms that can further refine the accuracy of market predictions and the efficiency of production processes. Additionally, the ethical considerations and potential impacts of AI on employment and industry practices warrant careful examination to ensure sustainable and responsible advancements in this field.

In conclusion, the strategic application of AI and big data analytics heralds a new era for cross-border e-commerce, offering unprecedented opportunities for growth and innovation. As we move forward, it is imperative for businesses, policymakers, and researchers to collaboratively foster an environment that nurtures technological advancements while addressing their socio-economic implications, thereby paving the way for a more prosperous and inclusive global market.
